# Reinforcing Efficiency of Recycled Carbon Fiber PLA Filament Suitable for Additive Manufacturing

**DOI:** 10.3390/polym16152100

**Published:** 2024-07-23

**Authors:** Loredana Tammaro, Alfonso Martone, Barbara Palmieri, Carmela Borriello, Sabrina Portofino, Pierpaolo Iovane, Fabrizia Cilento, Michele Giordano, Sergio Galvagno

**Affiliations:** 1 Nanomaterials and Devices Laboratory, Department for Sustainability, Sustainability Materials Technology and Processes, ENEA, P.le E. Fermi, 1, 80055 Portici, Italy; carmela.borriello@enea.it (C.B.); sabrina.portofino@enea.it (S.P.); pierpaolo.iovane@enea.it (P.I.); sergio.galvagno@enea.it (S.G.); 2 Institute of Polymers, Composite and Biomaterials (IPCB), National Research Council, P.le E. Fermi, 1, 80055 Portici, Italy; alfonso.martone@cnr.it (A.M.); fabrizia.cilento@ipcb.cnr.it (F.C.); michele.giordano@cnr.it (M.G.)

**Keywords:** recovered carbon fiber, polylactic acid, composites, additive manufacturing, FFF, mechanical properties

## Abstract

The use of 3D printing technology for manufacturing new products based on sustainable materials enables one to take advantage of secondary raw materials derived from recycling. This work investigates the structural performances of 3D printing composite filaments based on polylactic acid (PLA), as a matrix, reinforced by recycled carbon fiber (rCF). Carbon fibers were recovered from industrial scraps by a patented thermal process and used to produce thermoplastic composite filaments for additive manufacturing without any additional treatment and additives. The influence of the recovered carbon fiber (rCF) content on the thermal properties, mechanical properties and microstructure of the composites was studied in the range of 3–20 wt%. The recorded TGA curves exhibited a one-stage weight loss within the temperature range 290–380 °C for all samples and the residual rCF content was in good agreement with the theoretical fiber loading. The Young modulus of the extruded filaments strongly increased below a critical content (5 wt%), while at higher content the improvement was reduced. An increase in the storage modulus of 54% compared to neat PLA 3D printed sample resulted in a printed specimen with a higher rCF content. SEM images highlighted a strong rCF prevailing alignment in the direction of the extrusion flow, creating almost unidirectional reinforcement inside the filament. These findings suggest that homogeneous composite filaments reinforced with well-dispersed recycled CF without additional chemical modification and additives are suitable materials for additive manufacturing. The effect of rCF topological distribution within the material on the mechanical performances has been discussed, highlighting that the isolated fibers could efficiently transfer loads with respect to the percolated 3D network and have been correlated with the microstructure.

## 1. Introduction

Carbon fibers (CFs) are inorganic fibers about 5 to 10 μm in diameter and composed mostly of carbon atoms, with outmost properties, such as high stiffness, high tensile strength, high strength to weight ratio, high chemical resistance, high-temperature tolerance, and low thermal expansion, but extremely light in weight.

Their properties make them quite similar to steel, but since carbon fibers are five times lighter than steel, their weight is similar to that of plastics. Due to their high price as compared to other types of reinforcing fibers such as glass, basalt or plastic fibers [1], CFs are usually used in high-quality products.

In particular, they are largely used as reinforcing agents in the manufacturing of polymeric composites, the binding polymer being a thermoset resin (such as epoxy) or a thermoplastic polymer (such as polyester, vinyl ester, or nylon). The properties of the final product, carbon fiber-reinforced composite (CFRC), will then be affected by the nature of polymers and by the type of additives introduced into the binding matrix (resin).

CFRC have increasingly gained attention over the past years because of their high strength, light weight and excellent mechanical properties, thus increasing their application in many sectors, such as automotive, aerospace, military, wind turbines, and other advanced civil product sectors [2,3,4,5].

For this reason, the global demand for carbon fiber in 2023 reached 115,000 tons; aerospace (19.1%), wind blade (17.4%), sports leisure (16.3%), pressure vessels (12.2%), molding and compound (9.6%), C/C composites (8.7%), automotive (7.8%) and construction (4.2%) are the main fields in the market [6].

Currently, these high numbers and the growing use of CFRCs have affected the amount of CF waste. The staggering generation of CF waste reached 62,000 tons in 2020 [7] and, if no action is taken, the global CF waste output mainly from the aircraft sector and the wind turbine industry is estimated to be nearly 500,000 tons by 2035 [8]. There are two main types of carbon fiber waste. The first type results from dry fibers, unused, expired and off-spec materials, cutting waste and production scraps (cured and pre-impregnated), made of both thermoset and thermoplastic materials. The second type of waste comes from end-of-life products made of carbon fiber-reinforced composites, which are far more difficult to recycle.

The management of end-of-life products (EOLPs) represents a key point in the logic of a sustainable industry, in accordance with the principles of the circular economy, and the development of the proper recycling routes must be driven by both economic and environmental concerns; accordingly, the recycling processes of CRFC waste must be able to give back fibers which could be later reused for the manufacturing of new composites [9,10]. Therefore, the recycling process is one of the best ways to reduce the environmental impact and meet the world’s growing demand for this material in several industrial applications.

A massive number of methods, mainly based on mechanical, thermal or chemical processes, have been studied and established for the recycling of CFRC, which can be selected according to the type of material to be recycled and the application in which it will be reused [11,12,13]. Generally, the recovered fibers are fragmented into short lengths, because of size reduction or breakage of the waste before, during or after the treatment, so the recyclate is in a filamented, random, low-density-packing (fluffy) form. Therefore, the existing manufacturing processes—developed for virgin materials, typically available as sized tows—must be adapted to the unique recycled-fiber form or new methods for reinforcing and applying the recycled fibers must be found. In any case, even if the type of recycling process can influence the properties of the recovered fibers, they have been shown to retain most of their virgin mechanical properties, usually in a percentage higher than 90% [11].

Among the remanufacturing processes applied to the recovered fibers, it is worth mentioning the injection molding, extrusion/compression molding and 3D printing. In particular, additive manufacturing (AM), also known as three-dimensional (3D) printing, is a collection of methods based on layer-by-layer deposition manufacturing technologies in which computer-aided design (CAD) is used to build physical 3D objects [14,15,16].

In the literature, different techniques are proposed for the additive manufacturing of carbon fiber-reinforced polymers, such as fused filament fabrication (FFF), stereo lithography (SLA), selective laser sintering (SLS) and laminated object manufacturing (LOM). Among these techniques, FFF, or filament freeform fabrication, is a 3D printing process that uses a continuous filament of a thermoplastic material. FFF is the most studied and applied technique for the fabrication of composite tools due to its superior mechanical properties and cost and timesaving advantages [17].

Many thermoplastic polymers, such as acrylonitrile–butadiene–styrene polymer (ABS) or polylactic acid (PLA), as well polyamide (PA) or polypropylene (PP) have been already applied to the production of fiber-reinforced polymer filaments [18], and recently a new class of polymers, named technical polymer, like polyphenylene sulfide (PPS) and polyether ether ketone (PEEK) [19] has started to be used for the production of fiber-reinforced polymer filaments.

PLA is one of the best candidate materials in FFF [20] because it has good printability, good mechanical properties, biocompatibility and it is totally biodegradable. Water and carbon dioxide are produced as end products after decomposition of PLA. Therefore, it is non-toxic and does not cause environmental pollution. However, PLA in pure form is brittle and has low toughness and flexibility. To overcome the drawbacks, composites of PLA are used. The incorporation of CF in the PLA matrix in AM leads to the improvement of mechanical properties, such as elastic modulus and tensile strength [21].

Moreover, the control of elastic-dissipative behavior of reinforced materials is a key aspect to promote their technological applications; for example, a mechanical reinforcement and an increase in dissipative capacity has been proven to be effective for the realization of joints between dissimilar materials [22].

The application of recovered carbon fibers (rCFs) in additive manufacturing, mainly FFF, allows the reuse of rCFs for the manufacturing of new CFRC materials [23].

In this context, carbon fibers have been recovered from industrial scraps by a patented thermal recycling process [24,25] and reused as reinforcing agent for the fabrication of composite 3D printing filaments.

Dried powders based on neat PLA and PLA composite powders containing 3, 5, 10 and 20 wt% of rCFs were extruded into filaments by a single screw extruder at a temperature of 180 °C. Neither additional treatment after the CF recycling process nor additive were used for the manufacturing of all filaments.

All filaments were studied in terms of thermal properties via thermogravimetric analysis (TGA) and differential scanning calorimetry (DSC), and mechanical properties by tensile tests.

The TG curves of all filaments exhibited thermal degradation as a one-stage weight loss within the temperature range of about 290–380 °C. The residual rCF content compares well with the theoretical fiber loading, suggesting a homogeneous fiber distribution along with the produced filaments. Similar trends of DSC thermograms were found for all composite filaments: Tg and Tm temperatures are almost unaffected by the addition of rCF to PLA. The Young modulus of the extruded filaments strongly increased below a critical rCF content (5 wt%), while at higher content the filler reinforcement is less efficient.

Studies on the morphology of the fractured filaments and printed specimens were carried out by Scanning Electron Microscopy (SEM). SEM images highlighted a strong rCF prevailing alignment in the direction of the extrusion flow, making an almost unidirectional reinforcement inside the filament.

All filaments were tested with the FFF 3D printing technique through a PRUSA printer. Three-dimensional test samples were printed and characterized via Dynamic Mechanical Analysis (DMA). An increase in the storage modulus (E′) of about 54% compared to neat PLA 3D printed samples resulted for samples with higher rCF content (PLA-rCF20).

Results show that even if the recycling procedure commonly involves a shortening of fiber dimensions, recovered carbon fibers can successfully work as a reinforcement phase within a polymeric matrix.

This study highlights that the effective reinforcement of rCFs is reduced with respect to pristine long fibers due to a combination of the reduced actual aspect ratio and to the removal of sizing related to the recycling procedure. As a matter of fact, improvement in the mechanical behavior in terms of Young’s modulus and storage modulus was succeeded by an increase in fiber content [26] and the effect of topological arrangement have been discussed in terms of reinforcing effectiveness. This work investigated for recycled carbon fiber the effect of dispersion state in the apparent fiber Young modulus through the study of the effective reinforcement. Two different reinforcing mechanisms were identified related to the arrangement of rCF within the hosting matrix. When the fibers are distributed as isolated fillers, they are capable of transferring load efficiently, while when the network becomes 3D connected (percolate regime), a loss in the reinforcing mechanism is recorded. It was found that the effective filler reinforcement was about 25 GPa in the case of isolated fibers while it drops to about 15 GPa when a percolated network is observed.

This finding has been correlated with the microstructure investigation. This gives carbon fibers recovered from industrial scraps a new use as reinforcing agent for the fabrication of composite 3D printing filaments.

## 2. Materials and Methods

### 2.1. Chemicals

Poly(L-lactide) 4043D (referred to as PLA) with 94% L-lactic acid content, number-average molecular weight (Mn) of 67 kDa and dispersity (Đ) of 2.2 (measured by GPC) (density, ρm: 1.24 g/cm^3^; Tg: 55–60 °C; Tm: 145–160 °C) was purchased from NatureWorks LLC (Minnetonka, MN, USA).

PLA pellets were dried in an oven for 4 hours at 80 °C before processing (as recommended by the supplier) to reduce the molecular weight loss attributed to excessive hydrolysis.

Carbon fibers (rCFs) were recovered from PPS/CF (Cetex TC1100, Toray Advanced Composites, Nijverdal, the Netherlands) composite scraps materials derived from sheet lamination-based additive manufacturing post-processing kindly supplied by Aerosoft S.p.A (Capua, Italy). The data sheets indicate a resin content of nearly 34 wt%.

Recovery tests were performed on about 5 g of sample/recovery cycle in a tubular furnace Lenton (LTF 12/100/610 model) using the patented carbon fiber-recovery process developed by ENEA [24].

The recovered rCF fibers have a length in the range of 5–400 μm, a diameter of 5–7 μm and a density of 1.75 g/cm^3^. The density of the carbon fibers was determined according to the standard ISO 10119:2020—Method A [27]. The obtained value was compared to that stated in the data sheet of the starting material (PPS/CF, Cetex TC1100, Toray Advanced Composites, Nijverdal, The Netherlands).

**Figure 1:**
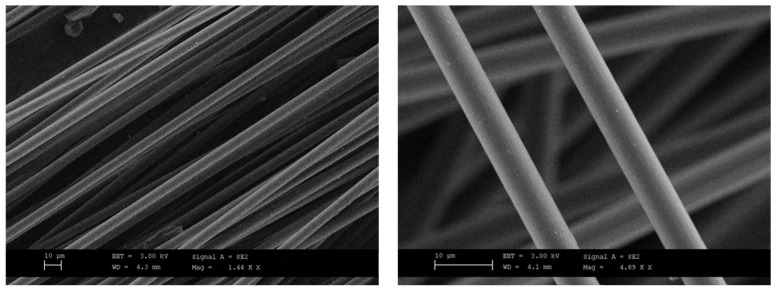
SEM images at different magnification of recovered carbon fibers.

The fibers were morphologically characterized by scanning electron microscopy (Figure 1); the figures show that the process allows one to gain undamaged recovered fibers, free from any residual carbon coating.

According to the patented process adopted for the recovery [24], rCFs retain 90% of the mechanical properties (in terms of tensile strength and Young modulus) of the virgin CFs.

### 2.2. Preparation of PLA/rCF Composites

Dried PLA pellets were converted into powder form using a grinding apparatus IKA-Werke model M20 (Staufen, Germany) operating at a fixed speed of 20.000 rpm.

PLA powders were then mechanically mixed with recovered carbon fibers. Composites with 3, 5, 10 and 20 wt% rCF were prepared along with neat PLA. Table 1 highlights the designations used in this study for the various composite compositions.

### 2.3. Filament Extrusion of PLA and PLA/rCF Composites

To verify the application of the recovered carbon fiber in the additive manufacturing (AM) process, the polymer–carbon fiber composites were extruded in the form of filaments and then used for 3D FFF.

The capability of a 3D model to be printed successfully (printability) on a specific AM machine first depends on the nature of the filaments and then the printing process parameters. Therefore, several tests were performed for improving the processability (extrudability) of the PLA/rCF composite mixtures, thus producing filaments with a specific and consistent size (length and diameter) and strength throughout the whole filament.

The composite mixtures listed in Table 1 were used to produce square specimens (60 × 60 × 2 mm) by a manual hydraulic press (SPECAC, Orpington, UK) operating at 180 °C. Then, the specimens were cut into small pieces, ground into powder through an IKA machine and then dried in a vacuum oven for 4 h at 80 °C before processing.

The dried composite powders were then extruded into filaments by a single screw extruder (Felfil Evo, Turin, Italy) at a temperature of 180 °C and rotational speed of 10 rpm, with a nozzle diameter of 1.75 mm.

With the need of improving the dispersion of the fibers into PLA samples with high rCF content, a three-step extrusion cycle of the filaments PLA-rCF10 and PLA-rCF20 was repeated two times. The cycle involved (a) filament grounding, (b) powder drying and (c) powder extruding. The cycle applied on PLA-rCF10 and PLA-rCF20 led to an effective improvement in the dispersion of the fibers in the PLA matrix without affecting the thermal stability of the resulting three-times extruded powders. Filaments extruded with diameters between 1.65 and 1.85 mm were then selected for the subsequent 3D printing process. Blank PLA was used as reference sample. PLA filaments were manufactured by extrusion from dried PLA pellets by means of the same extrusion parameters used for PLA/rCF composites.

### 2.4. 3D Printing of PLA/rCF Filaments

In compliance with 3D printer producers, recommendations for printing of composite materials filled with high-content abrasive filler, i.e., carbon fibers, suggest the use of a nozzle with a diameter of 0.8 mm to avoid clogging during the extrusion. Furthermore, the samples were printed using a hardened steel nozzle due to the concern of carbon fiber abrasion. In addition, a higher temperature of the nozzle is preferred to improve the viscosity of the melt and facilitate extrusion. Indeed, a temperature of 220 °C, higher than the 200–210 °C commonly used for neat PLA printing, was used.

The produced filaments were fed into a PRUSA I3MK3S (Prusa Research, Jesolo, Italy) FFF 3D printer to print tensile samples for characterizing the mechanical performance of the considered materials. Rectangular test specimens were designed using SolidWorks 2022 software. The design was exported into Standard Tessellation Language (.STL) and processed by the last version of Prusaslicer software (Prusa Slicer 2.7.0.) to obtain the instruction files for the 3D printer (.gcode). Samples of 40 × 10 × 1.5 mm (L × W × H) dimensions were printed using a hardened steel nozzle with a diameter of 0.8 mm.

Optimal printer settings were selected based on the initial printouts of the extruded filament. The 3D models were printed with a high resolution (0.2 mm layer height), 100% (the highest infill printing value) rectangular infill, filling angle 45° and the speed while extruding was set to 35 mm/s. Different combinations of the 3D printing parameters were tested and the main optimized process parameters used for the manufacturing of all composite samples regardless of rCF content are listed in Table 2.

Photos of the extruder, examples of an extruded filament and printed samples are shown in Figure S1. Details of the process from material to printing samples are schematically represented in the flow chart in Figure S2.

### 2.5. Characterization and Analysis of 3D PLA/rCF Composites

Differential scanning calorimetry (DSC) and thermogravimetric analysis (TGA) were performed with a simultaneous Thermal Analyzer –STA 449 F3 Jupiter^®^ (Netzsch, Germany) to detect the thermal behavior of the neat PLA and PLA-rCF composite filaments. The tests and data analysis were carried out according to international ASTM standard, E1131-08 [28] for TGA and DSC, respectively.

The TGA analysis was performed in triplicate and the residual mass of the sample was evaluated and the experimental values compared with the theoretical ones to verify the homogeneity of rCF dispersion into the PLA matrix. The derivative thermo-gravimetric (DTG) analysis was used to measure the rate of change of mass which gives information about the quantity of sample loss at the degradation temperature. Approximately 6–10 mg of the filament was examined from 30 to 900 °C at a heating ramp rate of 10 °C/min under a nitrogen gas flow of 50 mL/min.

The printed samples were kept in liquid nitrogen for 5 min before subjecting them to fragile fracture. Three-dimensional printed fractured specimens were used for SEM analysis and mechanical tests without any further treatment.

Dynamic mechanical analysis (DMA) was performed with a Dynamic Mechanical Analyzer Q800 (TA Instruments, Crawley, UK) in the single cantilever mode (SC). Samples of the rectangular shape 40 mm in length, 10 mm in width and about 1.5 mm in thickness were tested. The behaviour of the polymer in the temperature range of 30 °C to 100 °C was investigated considering a heating rate of 3 °C/min, a strain amplitude of 25 μm, and a frequency of 1 Hz. Data were elaborated according to the ASTM D790 standard for the flexural behaviour of unreinforced and reinforced plastics [29].

Tensile tests on filaments were performed with the Dynamic Mechanical Analyzer Q800 using the tensile clamp. Samples of cylindrical shape with nominal size of 20 mm in length and 1.75 mm in diameter were tested at room temperature with a displacement rate of 100.00 µm/min up to a final displacement of 2000.00 µm. Data were elaborated according to the ASTM D638 standard for plastic tensile strength tests [30].

Surface morphology of the section of fractured samples was analyzed using scanning electron microscopy (SEM). Micrographs were collected by a Phenom SEM Thermo Fisher Scientific (Waltham, MA, USA) with an operating voltage of 15 kV.

## 3. Results and Discussion

### 3.1. Thermal Properties

The influence of the different loadings of rCFs on the thermal behavior of the produced filaments was evaluated through differential scanning calorimetry (DSC) and thermogravimetric analysis (TGA). The DSC curves were used to determine the thermal properties, including the glass transition temperature (Tg) and melting temperature (Tm).

Thermal stability of all PLA-rCF extruded filaments was determined by TGA analysis and compared with neat PLA filament. The degradation onset and endset temperatures and the recycled carbon fiber content (e.g., residue mass after the end of experiments) were obtained from the thermogravimetric (TG) curves.

**Figure 2:**
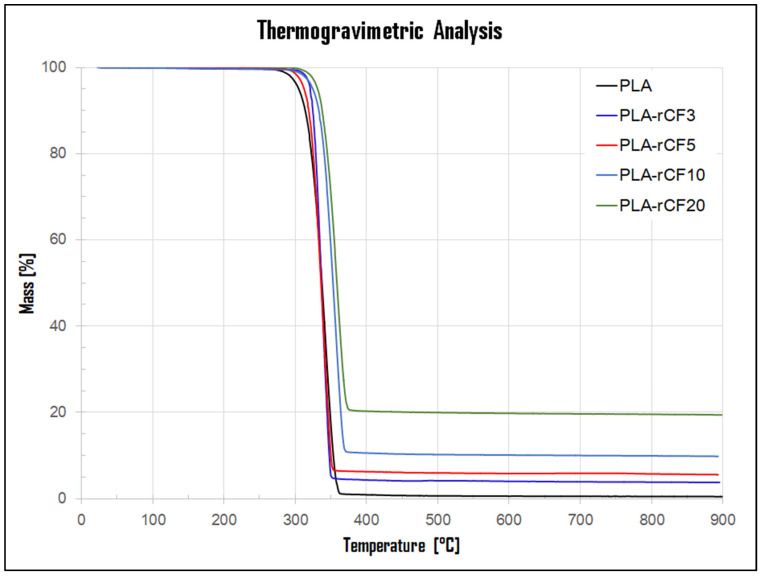
TGA curves of neat PLA and PLA-rCF3, PLA-rCF5, PLA-rCF10, PLA-rCF20 filaments.

The TG curves of all filaments are depicted in Figure 2 and exhibited thermal degradation as a one-stage weight loss within the temperature range of about 290–380 °C. The PLA material undergoes full degradation, while the PLA-rCF composites show residual mass at 450 °C, indicating the presence of incorporated recycled carbon fibers. According to Table 3, the rCF content (residue mass) in the various filament composites, performed in triplicate, compares well with the feed, suggesting a homogeneous fiber distribution along with the produced filaments by using the proposed mixing methodology.

**Figure 3:**
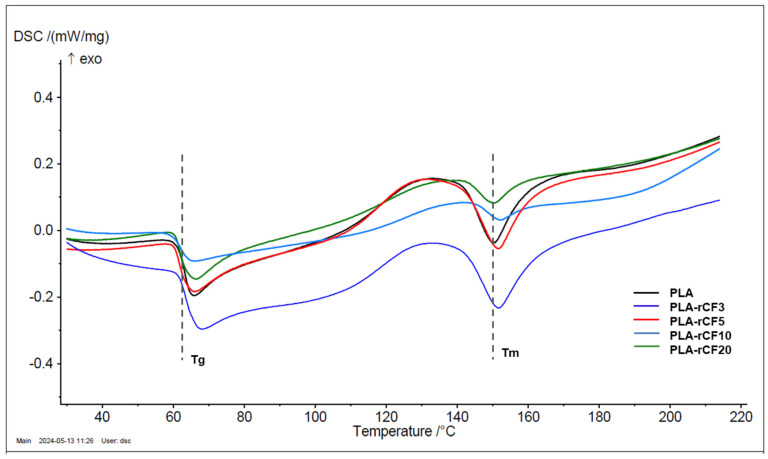
DSC thermograms of neat PLA and PLA-rCF3, PLA-rCF5, PLA-rCF10, PLA-rCF20 filaments.

Almost all PLA-rCF filaments show onset and endset values higher or close to neat PLA. A comparable trend resulted also for the 5% and 50% mass loss temperatures. DSC curves of the neat PLA filament and the composite filaments are reported in Figure 3, whereas data are summarized in Table 1. The DSC thermogram of the neat PLA filament shows a stepwise transition at around 63 °C, ascribed to PLA glass transition temperature (Tg), followed by an intense endothermic peak at 150 °C, due to PLA melting [31]. A broad exothermic transition at about 130 °C can be ascribed to a melt crystallization [32], typical of semi-crystalline polymers characterized by a low crystallization rate, prior to an endothermic peak. A very similar trend is observed for all the composite filaments. The Tg and Tm temperatures are almost unaffected by the rCF addition to PLA in all the filaments, as reported in Table 3.

Generally, the fiber volume fraction is calculated according to ASTM D2584 [33] as Equation (1)

(1) $$Vf=\frac{\rho m*Wf}{\rho m*Wf+\rho f*Wm}$$

where *Vf* is volume fraction of fibers, *Wf* is weight of fibers, *Wm* is weight of matrix, *ρf* is density of fibers, *ρm* is density of matrix.

The values of *Vf* for all PLA-rCF composites were calculated according to Equation (1) and listed in Table 3.

### 3.2. Morphological Characterization

The morphology of extruded filaments and 3D printed samples was evaluated using a tabletop scanning electron microscope (SEM). rCF dispersion and alignment of the section of composite samples was analyzed after fragile fracture.

**Figure 4:**
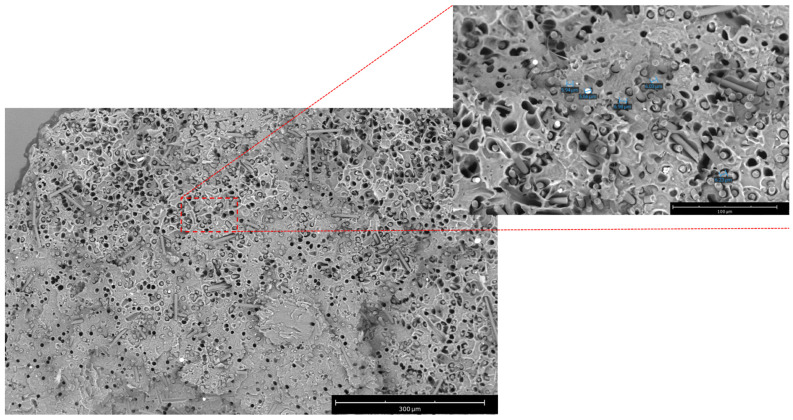
SEM micrograph (scale bar: 300 µm) at 520× magnification (inset at 1600×, scale bar: 100 µm) of PLA-rCF20 fractured filament analyzed in section.

The filament with the higher rCF content was analyzed in section by SEM analysis to study the rCF distribution and alignment. The cross-sectional images of the PLA-rCF20 filament reveal an even distribution of rCF with fiber pullout cavities and fiber breakage. SEM images showed a strong rCF alignment along with the longitudinal direction of the extrusion flow (Figure 4), as also reported by Giani et. al. [34]. The unidirectional alignment of the fibers was well visible in the sample with 20% rCF content; this could be attributed to the shear forces acting on the molten PLA during the extrusion process that increase as rCF concentration increases.

**Figure 5:**
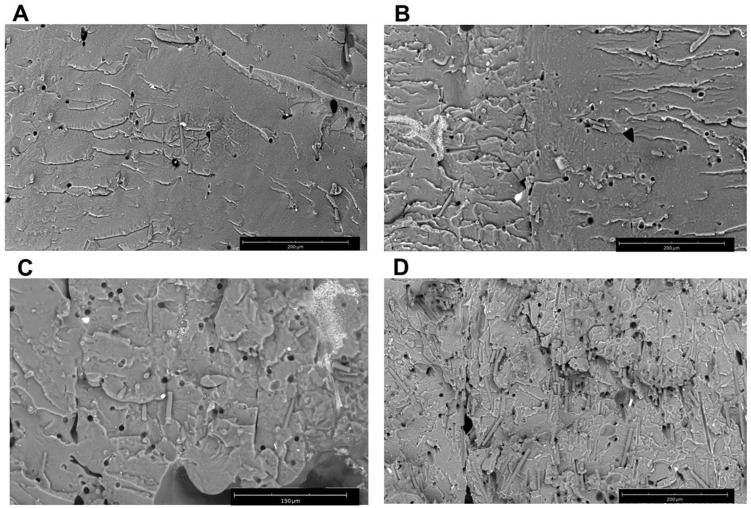
SEM images (magnification 750×) of the cryo-fractured 3D printed specimens of the compounds (**A**) PLA-rCF3 (scale bar: 200 µm); (**B**) PLA-rCF5 (scale bar: 200 µm); (**C**) PLA-rCF10 (scale bar: 150 µm); (**D**) PLA-rCF20 (scale bar: 200 µm).

Micrographs of the cryo-fractured printed specimens with the different rCF contents are reported in Figure 5. The rCF can be clearly seen as fibers protruding from the fractured surface. Indication of fiber pullout is seen by the presence of many holes with similar geometrical shape and dimension as the carbon fiber cross sections.

Additionally, holes in the fracture surface are visible and increase in number with increasing rCF content. Moreover, no agglomerates of fibers at low rCF concentration appear unlike the sample with the higher fiber content (PLA-rCF20, Figure 4) showing few fiber agglomerations. Furthermore, most of the fiber surfaces visible are uncoated with the matrix polymer, indicating a weak matrix–filler interface.

### 3.3. Filament Mechanical Characterization

**Figure 6:**
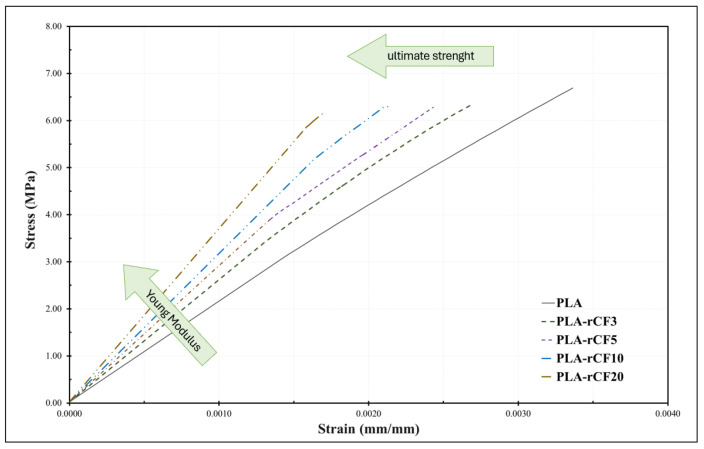
Quasi static tensile test on PLA-rCF filaments.

Figure 6 shows the Young modulus of PLA-rCF filaments at each filler content. The elastic modulus was computed in the elastic regime in the strain range between 0.03% and 0.10%. According to results, the tensile modulus, $E_{c}$, increases as a function of the rCF content (wt%); the higher the filler content, the higher the modulus. Moreover, the critical strain at the failure is reduced by increasing the filler content, leading to a fragile behavior combined with a slight strength decrease.

As known, the increment of CF percentage in composite structure compromises its ductile nature and begets brittleness. Indeed, the inclusion of CF in the polymeric matrix triggers crack formation upon application of load. With time, this turns into crack propagation, and a chain reaction rages through all the cracks in the vicinity, resulting in the brittle nature [35,36,37,38].

In the present case, micro-failures form at the fiber ends or at the contacting points (when the network becomes percolated) that propagate. The higher the contact number (i.e., the amount of rCF), the higher the probability that cracks initiate and propagate inside the materials, triggering the behavior from ductile (bare polymer) to fragile, with a critical deformation that reduces at higher rCF content, even if the stiffness is increased as an effect of the rise of stress-transfer capability.

In the case of recycled carbon fiber/PLA composites, the strength achieved is comparable to those presented by Omar et al. [38]. The recycling process erases the sizing onto the carbon fiber surface, threatening the chemical bonding between polymer and fiber.

**Figure 7:**
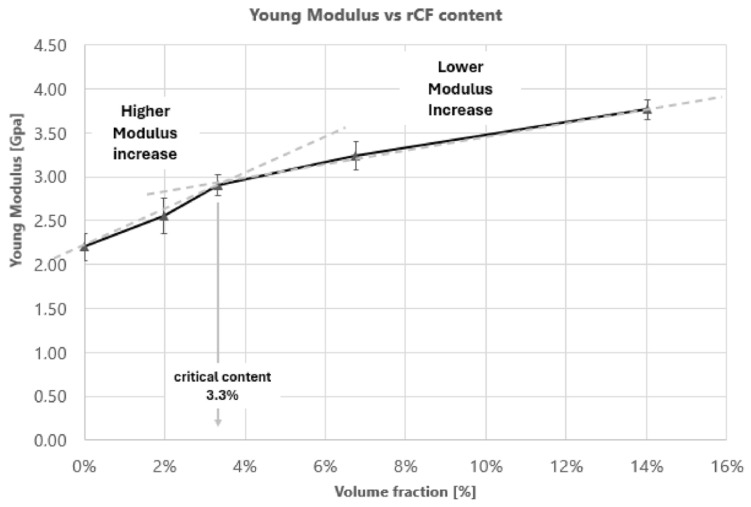
Young Modulus results vs. rCF volume fraction.

The dependence of materials’ stiffness identifies two different behaviors, below a critical content (3.3% vol) and above the critical volume content. Experimental data show that below the “percolation onset” the filler is able to reinforce the matrix with higher efficiency while above that content the shear transfer between hosting matrix and filler is less efficient (Figure 7).

### 3.4. Viscoelastic Characterization

**Figure 8:**
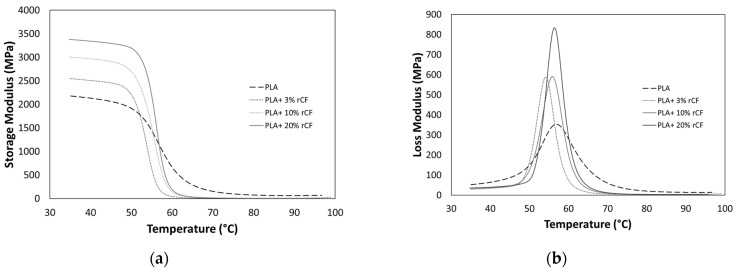
(**a**) Storage modulus (E′) and (**b**) loss modulus (E″) of 3D printed samples at different rCF content.

The viscoelastic behavior of the 3D printed samples at temperatures up to 100 °C at different rCF content is depicted in Figure 8. Figure 8a reproduces the storage modulus (E′), which is related to the elastic response of the material, while Figure 8b reproduces the loss modulus (E″) which is related to the capability of the material of dissipate energy. DMA experiments were conducted in the linear viscoelastic deformation range (0.1%) to determine the glass transition temperature and viscoelastic performances. The results of DMA tests at the temperature of 35 °C and at the loss modulus peak temperature are summarized in Table 4.

According to Cox’s shear lag model [39], the effective tensile modulus of a matrix reinforced with short fibers should be defined by an efficiency factor, η, that is given by the ratio between the effective reinforced modulus of the filler and its intrinsic modulus. The model describes the effect of the fiber aspect ratio upon its reinforcing efficiency in a composite. The complete system of equations is reported in Equation (2).

(2) $$E_{c}=E_{\eta }\cdot \phi +E_{m}\cdot \left(1-\phi \right)\;E_{\eta }=\eta \cdot E_{NT}\;\eta =1-\frac{\tanh\left(K\cdot AR\right)}{\left(K\cdot AR\right)}\;K=\sqrt{\frac{2E_{m}}{E_{r}\cdot (1+\upsilon_{m})\cdot \ln(1/f_{r})}}$$

**Figure 9:**
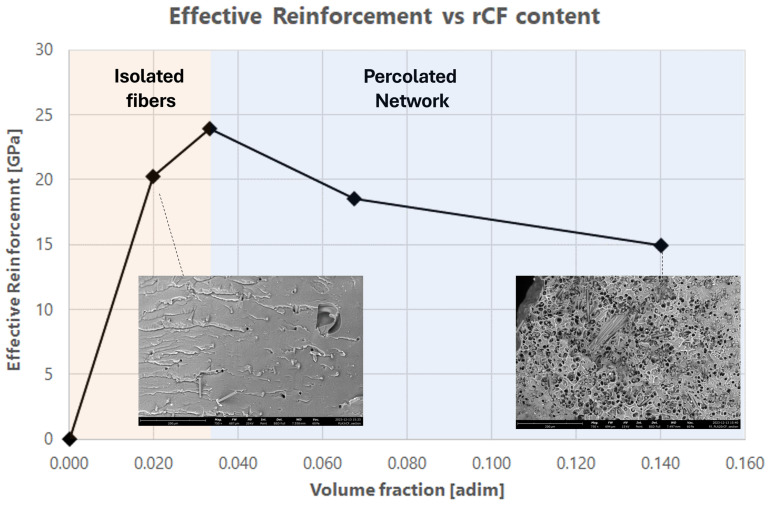
Effective reinforcement modulus, $E_{\eta }$, as a function of the rCF volume (%).

$E_{c}$ is the tensile modulus of produced reinforced filament, $E_{\eta }$ is the effective reinforcement modulus, $E_{m}$ is the neat matrix modulus and ϕ is the rCF volume (%). The critical percolation threshold was evaluated according to geometrical critical percolation for 1D filler [40]. Considering as critical condition the value of rCF volume (%) at which $E_{\eta }$ decreases, it is possible to obtain the experimental critical value of the Aspect Ratio (AR). As highlighted in Figure 9, the $\upsilon_{crit}$ is equal to 0.033, so according to (3) the ${AR}^{exp,crit}$ obtained is equal to 15.

(3) $$\upsilon_{crit}=\frac{1}{2AR}\to {AR}^{exp,crit}=15$$

The composite tensile modulus as a function of rCF content (Figure 9) exhibits a highly nonlinear trend with a maximum value (≈0.033 vol.%), after which a decreasing function is shown.

rCF filler contributes to the composite mechanical stiffness until an effective modulus that decreases with the filler content above $\upsilon_{crit}$ that represents the passage between the isolated fiber area to the percolated network one.

The reinforcement effect of a filler plunged in a hosting matrix is related to its capability to transfer stresses between the matrix and the filler. The stresses are transferred by shear along the filler; in the case of unidirectional fillers, the mechanism is dominated by shear lag. An accurate analysis of the shear lag phenomenon related to 1D reinforcement surrounded by a polymeric matrix has been carried out by Nairn [41], who started with the representative volume element defined by Carman and Reifsnider [42]. The reinforcement effect depends on the filler aspect ratio. Actually, the latter analysis is correct when the fiber is in dilute regime, i.e., isolated in the surrounding matrix, when the content of filler is increased; nevertheless, this assumption fails. The contact between reinforcement acts as a defect, reducing the actual aspect ratio of the filler and therefore the reinforcing efficiency. Philipse [43] exploited the percolative behaviour of rod filled systems allowing one to correlate the number of contacts (<c>) to the volume occupied by the filler (Equation (4)).

(4) $${AR}_{eff}=\frac{AR}{1+<c>}$$

Martone et al. [40] developed a numerical procedure to model the progressive reduction of AR as a function of filler content in percolated networks.

Similarly, in the case of recycled carbon fiber the progressive loss in effective reinforcement was measured.

Results, summarized in Table 4, indicate that the presence of the filler slightly increases the storage modulus of the composite, as observed with the tensile test on the filaments. For example, in cases of the PLA being reinforced with 20 wt% rCF, the storage modulus (E′) increases by 54% compared to the 3D printed sample using the unreinforced filament. The dissipation capacity, measured by the loss modulus (E″) at 35 °C decreases with the filler content, indicative of a more rigid system. In cases of 20 wt%, the loss modulus is higher than other reinforcement contents, which could be linked to agglomeration formation.

In fact, as known, improvement of mechanical properties of fiber-reinforced composites depends on the content of fillers. On the other hand, when the content of fillers is increased beyond a certain limit, then the micro-spaces between the fillers increase, resulting in agglomeration of them. This reduces the bonding strength between the matrix and fibers, reducing the mechanical strength of the composites [44].

**Figure 10:**
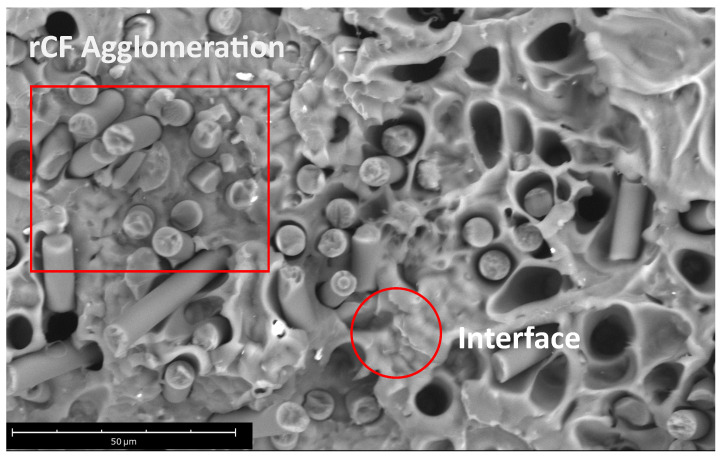
Details of SEM micrograph at 3300× magnification of PLA-rCF20 fractured filament analyzed in section.

Analyzing the results at the E″ peak that corresponds with the glass transition temperature of the materials, the reinforcement content does not affect the storage modulus, while an enhancement on the E″ is evident. In fact, the higher the filler content, the higher the dissipative capacity of the systems. Under the 3 wt% content, the increases in the loss modulus (about 66%) are linked to a worse interface between the fiber and the matrix, which causes a relative sliding between them. Above the percolative threshold, in addition to this phenomenon, the presence of a percolated network contributes to increments in the dissipative capacity. In fact, in the case of 20 wt% of rCF, the value of E″ at peak is 136% higher than the unreinforced system. Saleh et al. [45] reported the agglomeration effect even for the sample with 8 wt% rCF and highlighted this feature by SEM analysis. Likewise, the presence of agglomeration in the sample with high rCF content and worse interface is highlighted by the SEM image reported in Figure 10.

## 4. Conclusions

In this work, PLA composites containing different recovered carbon fiber contents (3, 5, 10 and 20 wt%) were prepared and used for filament extrusion.

Based on experimental results, the following conclusions can be drawn:

(A) All composite filaments, although there was an absence of additional substances (e.g., stabilizer and/or compatibilizer) and treatment after CF recycling process, showed uniform dispersion of fibers, good extrudability and printability in a commercial FFF printer.
(B) Thermal analysis of extruded filaments showed an improved thermal stability of PLA-rCF filaments with respect to neat PLA filament, more evident at higher rCF content.
(C) The tensile modulus increases as a function of the rCF content (wt%), from 2180 MPa for neat PLA to 3360 MPa for PLA-rCF20 filament.
(D) SEM images showed no agglomerates of fibers at low rCF concentration unlike samples with higher fiber content (PLA-rCF20) for which a strong rCF alignment along with the longitudinal direction of the extrusion flow was detected in the related filament.
(E) Three-dimensional printed specimens from composite filaments were manufactured through a PRUSA 3D printer.
(F) An increase in the storage modulus of about 54% compared to the neat PLA 3D printed sample resulted for samples with higher rCF content (PLA-rCF20).
(G) In the 3D printed samples, two different reinforcing regimes were identified, which can be correlated with the percolation behavior related to the 1D reinforcement. In the isolated fiber regime, the reinforcement contributes to increasing the elastic modulus (E′), while in the percolated network the effectiveness of the reinforcement decreases and the dissipative capacity (E″) is improved.

## Figures and Tables

**Table 1:** Composition of PLA-rCF composites.

Sample Name	PLA Content (wt%)	rCF Content (wt%)
PLA-rCF3	97	3
PLA-rCF5	95	5
PLA-rCF10	90	10
PLA-rCF20	80	20

**Table 2:** Optimized technological parameters for 3D manufacturing.

Nozzle Temperature (°C)	Bed Temperature (°C)	Infill Type	Build Orientation (°)	Printing Speed (mm/s)	Layer Thickness (mm)	Infill Density (%)
220	60	rectangular	45	35	0.2	100

**Table 4:** Results of DMA analysis at 35 °C and at Temperature of E″ peak.

		35 °C			T Peak (E″)			
	T_g_, _DMA_	E′	E″	tanδ	T Peak	E′	E″	tanδ
	(°C)	(MPa)	(MPa)	(-)	(°C)	(MPa)	(MPa)	(-)
PLA	56.7 ± 1	2180 ± 45	52.3 ± 1	0.0241 ± 0.0005	56.8 ± 1	1141 ± 25	353.8 ± 10	0.3099 ± 0.008
PLA-rCF3	53.9 ± 1	2547 ± 52	29.8 ± 0.6	0.0117 ± 0.0002	54.3 ± 1	923 ± 22	588.6 ± 14	0.6377 ± 0.016
PLA-rCF10	55.5 ± 1	3006 ± 60	31.2 ± 0.6	0.0125 ± 0.0002	55.8 ± 1	1176 ± 28	590.6 ± 15	0.6086 ± 0.014
PLA-rCF20	56.1 ± 1	3360 ± 65	37.6 ± 0.7	0.0112 ± 0.0002	56.4 ± 1	1373 ± 32	834.0 ± 18	0.6073 ± 0.014

**Table 3:** TGA and DSC results for PLA and PLA-rCF composite filaments.

Sample	T_onset_ (°C)	50% Mass Loss T (°C)	5% Mass Loss T (°C)	T_endset_ (°C)	T_vmax_ (°C)	dW/dT T_vmax_ (%/°C)	Residual Mass (%)	Vf (%)	T_g_ (°C)	T_m_ (°C)
PLA	317.8	337.5	305.0	357.5	343.3	−2.65	0.46	0	63.3	150.3
PLA-rCF3	322.0	336.8	321.0	349.0	344.1	−4.91	3.7	2.3	63.6	151.6
PLA-rCF5	319.9	336.2	312.5	350.6	341.7	−3.54	5.5	3.6	61.7	151.4
PLA-rCF10	332.5	353.0	324.7	371.5	344.4	−3.56	9.8	6.8	62.2	152.2
PLA-rCF20	339.4	358.4	331.4	370.0	355.9	−3.70	19.4	14.2	61.8	150.0

T_onset_: onset of degradation temperature. T_endset_: endset of degradation temperature. T_vmax_: temperature of maximum degradation rate determined as the minimum point of the DTG derivative curve. dW/dT: speed of variation of the weight with respect to the temperature at T_vmax_. Vf: volume fraction of fibers. Tg: glass transition temperature. Tm: melting temperature.

## Data Availability

Data supporting this study are included within the article.

## Supplementary Materials

The following supporting information can be downloaded at: https://www.mdpi.com/article/10.3390/polym16152100/s1, Figure S1: (A) Single screw extruder (Felfil Evo, Italy); (B) extruded PLA-rCF20 filament; (C,D) 3D-specimens printed with PLA-rCF20 filament; Figure S2: Flow chart of the overall process from material to printing samples.
