# Supplementary material for: Reinforcing Efficiency of Recycled Carbon Fiber PLA Filament Suitable for Additive Manufacturing
Source: Polymers (Basel). 2024 Jul 23;16(15):2100. doi: 10.3390/polym16152100 (PMC11313939; doi:10.3390/polym16152100)
Supplement: Supplementary file 1 [file polymers-16-02100-s001.zip › polymers-3110411-supplementary.pdf]

Supporting Information

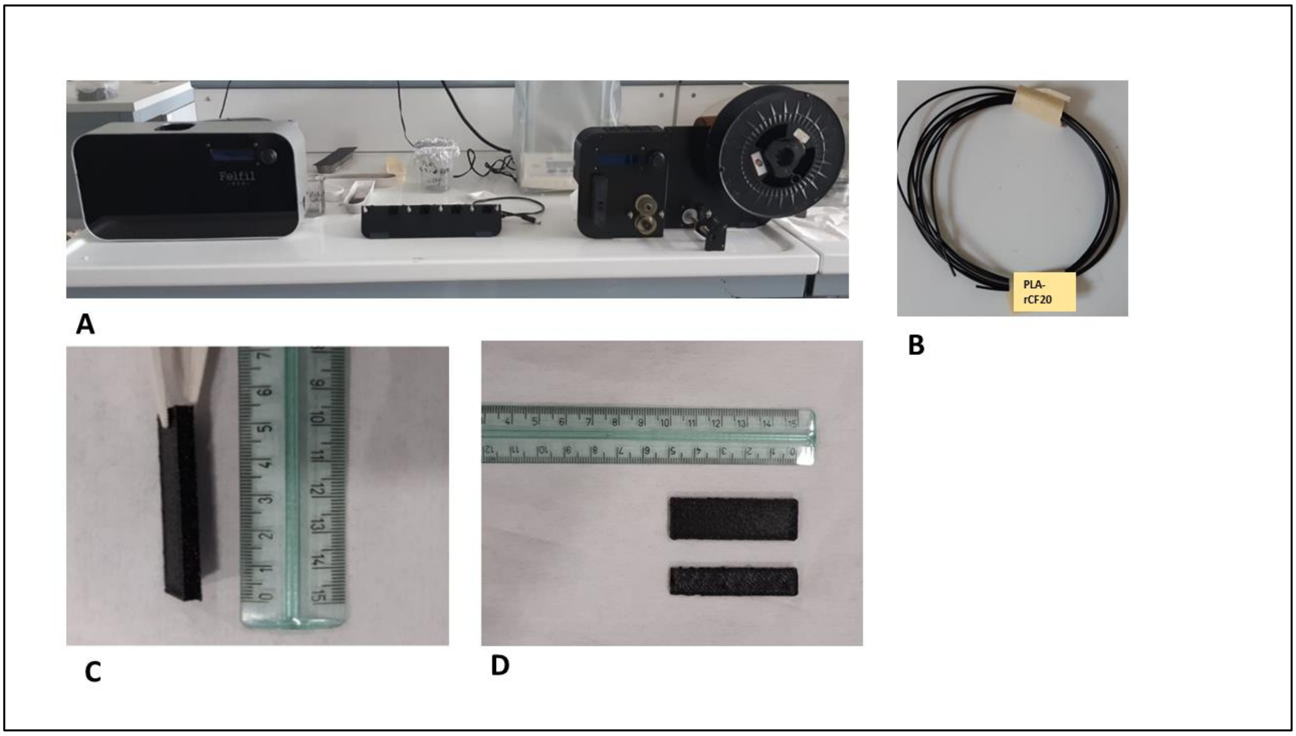

Figure S1. A) Single screw extruder (Felfil Evo, Italy); B) extruded PLA-rCF20 filament; C) and D) 3D-specimens printed with PLA-rCF20 filament

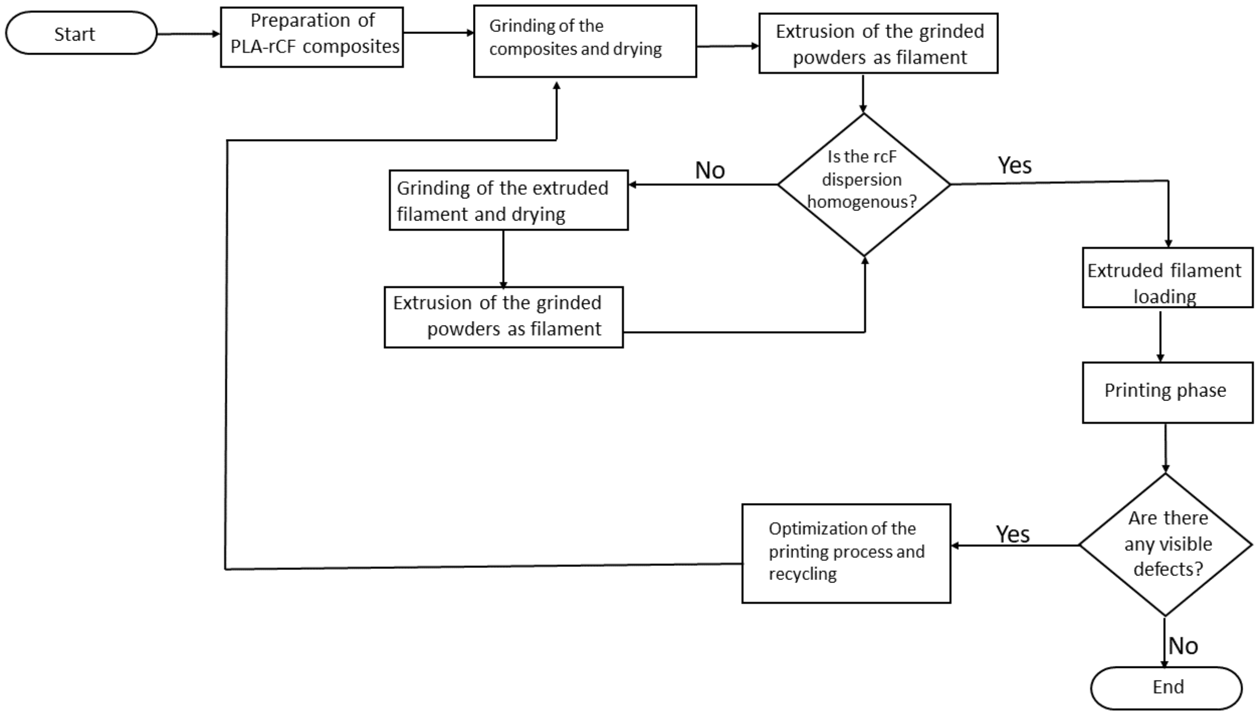

Figure S2. Flow chart of the overall process from material to printing samples.
